# β_2_-adrenoceptor blockage induces G_1_/S phase arrest and apoptosis in pancreatic cancer cells via Ras/Akt/NFκB pathway

**DOI:** 10.1186/1476-4598-10-146

**Published:** 2011-11-26

**Authors:** Dong Zhang, Qingyong Ma, Zheng Wang, Min Zhang, Kun Guo, Fengfei Wang, Erxi Wu

**Affiliations:** 1 Department of Hepatobiliary and Pancreas Surgery, First Affiliated Hospital, Xi'an Jiaotong University, Xi'an 710061, China; 2 Department of Pharmaceutical Sciences, North Dakota State University, Fargo, ND 58105, USA

**Keywords:** β-adrenergic antagonists, G_1_/S phase arrest, apoptosis, Ras

## Abstract

**Background:**

Smoking and stress, pancreatic cancer (PanCa) risk factors, stimulate nitrosamine 4-(methylnitrosamino)-1-(3-pyridyl)-1-butanone (NNK) and catecholamines production respectively. NNK and catecholamine bind the β-adrenoceptors and induce PanCa cell proliferation; and we have previously suggested that β-adrenergic antagonists may suppress proliferation and invasion and stimulate apoptosis in PanCa. To clarify the mechanism of apoptosis induced by β_2_-adrenergic antagonist, we hypothesize that blockage of the β_2_-adrenoceptor could induce G_1_/S phase arrest and apoptosis and Ras may be a key player in PanCa cells.

**Results:**

The β_1 _and β_2_-adrenoceptor proteins were detected on the cell surface of PanCa cells from pancreatic carcinoma specimen samples by immunohistochemistry. The β_2_-adrenergic antagonist ICI118,551 significantly induced G_1_/S phase arrest and apoptosis compared with the β_1_-adrenergic antagonist metoprolol, which was determined by the flow cytometry assay. β_2_-adrenergic antagonist therapy significantly suppressed the expression of extracellular signal-regulated kinase, Akt, Bcl-2, cyclin D1, and cyclin E and induced the activation of caspase-3, caspase-9 and Bax by Western blotting. Additionally, the β_2_-adrenergic antagonist reduced the activation of NFκB *in vitro *cultured PanCa cells.

**Conclusions:**

The blockage of β_2_-adrenoceptor markedly induced PanCa cells to arrest at G_1_/S phase and consequently resulted in cell death, which is possibly due to that the blockage of β_2_-adrenoceptor inhibited NFκB, extracellular signal-regulated kinase, and Akt pathways. Therefore, their upstream molecule Ras may be a key factor in the β_2_-adrenoceptor antagonist induced G_1_/S phase arrest and apoptosis in PanCa cells. The new pathway discovered in this study may provide an effective therapeutic strategy for PanCa.

## Introduction

Pancreatic cancer (PanCa) remains a lethal disease [1]. There is increasing evidence suggesting that many factors such as smoking, stress, chronic depression and a high-fat diet, with cardiovascular disease and stress patients may contribute to PanCa genesis and development but the underlying mechanisms are not clear [2-4]. Previous studies indicate that the enhanced tumour progression by smoking-stimulated nitrosamine 4-(methylnitrosamino)-1-(3-pyridyl)-1-butanone (NNK) production and stress-stimulated autonomic activation of nervous system [5,6]. The autonomic activation of nerve system results in the release of catecholamines from the adrenal gland and sympathetic nerve terminals. Further studies suggest that both NNK and constantly high level of catecholamines modulate the activity of multiple components of the tumour microenvironment and consequently promote tumour-cell growth via β-adrenoceptors [4,7-9].

β-adrenoceptors are members of the superfamily of G protein-coupled adrenergic receptors, which mediate actions of the endogenous catecholamines in a variety of target cells [10,11]. β_1_- and β_2_-adrenoceptors have been found to be expressed in the BxPC-3, MIA PaCa-2, and Panc-1 cell lines [12-14]. NNK functions as a β-adrenergic agonist and it has been shown that the binding of NNK or catecholamines to the β-adrenoceptors induce PanCa cell proliferation by activating the cyclic adenosine monophosphate (cAMP)/protein kinase A (PKA) pathways in PanCa cells [15]. The consequence of PKA signalling leads to the transcriptional activation of proteins involved in proliferation via cAMP response element binding protein (CREB), activator protein 1 (AP-1) or NF-κB [15-17]. Recent reports have shown that the agonists of the β_2_-adrenoceptor stimulate the activation of Ras and Src tyrosine kinases via the mitogen-activated protein kinase (MAPK) pathway in cancer cells and fibroblasts [18-20]. Ras activates several signalling pathways that lead to transcriptional activation of genes related to cell proliferation and antiapoptotic signalling cascades, including the Raf/MEK/extracellular signal-regulated kinase (ERK) and phosphatidylinositol 3-kinase (PI_3_K)/ATP-dependent tyrosine kinases (Akt)/Phosphatase and tensin homolog deleted on chromosome 10 (PTEN) pathways and NF-κB [17,21].

Previous studies suggested that β-adrenergic antagonists may suppress cell proliferation and invasion and induce apoptosis in PanCa [14,22], and also β_2_-adrenergic agonist can stimulate the production of cAMP and activation of G-protein effectors Gs [23]. However, the mechanism of PanCa cell death induced by β_2_-adrenergic antagonist is not clear. In this study, we determined the effects of β_2_-adrenergic antagonist ICI118,551 on PanCa tumor growth and the underlying mechanism *in vitro *and *in vivo*.

## Materials and methods

### Tumor tissues, Cell lines and cell culture

Forty-eight pancreatic carcinoma specimens were obtained from the Department of Hepatobiliary and Pancreas Surgery, the First Affiliated Hospital of Xi'an Jiaotong University. The protocol was approved by The Human Research Review Committee at the University Hospital. The human ductal pancreatic adenocarcinoma cell lines, MIA PaCa-2 and the BxPC-3 were purchased from the American Type Culture Collection (ATCC, Manassas, VA, USA). The MIA PaCa-2 cell line harbours an activating point mutation in codon 12 of the k-ras gene, whereas BxPC-3 does not have that k-ras mutation [24]. The cells were cultured in DMEM (Gibco, Grand Island, NY, USA) with 10% (v/v) heat-inactivated FBS (Gibco, Grand Island, NY, USA), penicillin (100 U/ml) and streptomycin (100 mg/ml)at 37°C with 5% CO_2 _and 95% relative humidity. Before each experiment, cells were seeded at a density of 5 × 10^4 ^cells/cm^2^.

### Immunohistochemistry staining

Immunohistochemical staining for β_1_-adrenoceptor and β_2_-adrenoceptor were performed using the SABC kit according to the manufacturer's instruction (Abfrontier, Korea). The sections were incubated with primary antibodies (Abcam, Cambridge, MA, USA) overnight at 4°C and then incubated with the appropriate biotinylated secondary antibody (Abcam, Cambridge, MA, USA) for 30 min at room temperature. Sections were then incubated with avidin/biotin complexes for 30 min at room temperature. Following washing in PBS, immunoreactivity was visualized by using DAB reaction followed by hematoxylin counter staining. For evaluation of protein expression, the staining intensity was graded as 0 (negative), 1 (weak), 2 (medium) or 3 (strong). The slides were graded by two investigators. The densitometry analysis of immunohistochemical staining was performed using the Image-Pro Plus 4.5 software.

### Cell cycle analysis

The cells after treatment were stained with propidium iodide (PI) and then analysed using flow cytometry for cell cycle according to the manufacturer's protocol. After stimulation for 24 h with ICI118,551 (100 μM) [13,14,25] (Sigma Chemical, St. Louis, MO, USA), MIA PaCa-2 and BxPC-3 cells were fixed in ice-cold 70% ethanol at 4°C. On the day of analysis, the cells were then centrifuged (400 × g for 5 min) and washed once in staining buffer. After centrifugation, the cells were resuspended in 100 μL of staining buffer. The cells were then treated with RNase A and incubated for 30 min at 37°C. After RNase A digestion, the cells were stained for 30 min at room temperature (protected from light) with 10 μg of PI in a final volume of 0.5 mL of staining buffer. The samples were then analysed by flow cytometry. The percentages of cells in G0-G1, S, and G2-M were calculated using the ModFit computer program.

### Animal studies and subrenal capsular assay

BALB/c athymic nude mice, 4 to 6 weeks old (Shanghai Experimental Animal Center (Chinese Academy of Sciences, China) were used for the subrenal capsule assay. PanCa xenografts were generated by implantation of MIA PaCa-2 and BxPC-3 cells into one flank of athymic nude mice. Each group contains n ≥ 8 male athymic nude mice (10 mice each group). On the day of the experiment, mice were anesthetized, and the left kidney was exposed. The same number of cells (1 × 10^8^) in 25 μl was then injected into each mouse under the left renal capsule. After three days, a 5 mg/kg/dose of ICI118,551 was intraperitoneally injected into the mice every day. After inoculation with the tumor cells, mice were observed and sacrificed on day ten, when their left kidneys were excised. The left renal capsule were fixed in 10% neutral buffered formalin and processed in paraffin. Sections were cut on a microtome and mounted on glass slides. For histopathology, routine H&E staining was carried out. Animal care and experiments were carried out in accordance with the guidelines of the Xi'an Jiaotong University.

### Electron microscopy

To evaluate the morphological features of cell death, the cells in the control group or cells treated with or without ICI118,551 (100 μM) were processed for transmission electron microscopy (TEM). Briefly, cells were pelleted by centrifugation and fixed with 2.5% glutaraldehyde in 0.1 M cacodylate buffer, pH 7.4 for 1 h at 4°C. After rinsing with cacodylate buffer, the specimens were fixed in 1% cacodylate-buffered osmium tetroxide for 2 h at room temperature, dehydrated in a graded series of ethanol, briefly transferred to propylene oxide and embedded in Epon-Araldite. Ultrathin sections (60-80 nm thick) were cut with a diamond knife, placed on formvar-carbon coated copper grids (200 mesh), stained with uranyl acetate and lead citrate and observed with a Jeol 100 SX TEM.

### Determination of cell apoptosis by hoechst 33342 fluorescent staining

The cell death was assessed using Hoechst staining. Briefly, cells were seeded in 24-well plates at 5 × 10^4 ^cells/well and treated with ICI118,551 (100 μM) for 24 h. After treatment, cells were stained with 10 μg/ml Hoechst 33342 dye (Sigma Chemical, St. Louis, MO, USA). Fixed cells were incubated overnight at 4°C and visualized under a 365 nm UV light microscope. Quantitative analysis was performed by counting the blue fluorescent (apoptosis positive) cells from three independent fields at 400× magnification. Values were expressed as the percentage of apoptotic cells relative to the total number of cells per field.

### Analysis of the apoptosis rate by Annexin V-FITC/PI

The apoptosis rate was measured using Annexin V-FITC/PI double staining followed by flow cytometry (FCM) according to the instructions (BD Biosciences, San Jose, CA, USA). After treatment with metoprolol (25-200 μM) and ICI118,551 (25-200 μM) for 24 h, MIA PaCa-2 and BxPC-3 cells were washed once with ice-cold PBS and resuspended in binding buffer at a concentration of 1 × 10^6 ^cells/ml. A total of 5 μL of Annexin V-FITC and 10 μL of 20 mg/mL PI were added to the cells, and the mixture was incubated for 15 min in the dark prior to the addition of 400 μl of PBS. Quantitative analysis of the apoptotic level was performed using a flow cytometer (BD Biosystems, CA, USA).

### Electrophoretic mobility shift assays (EMSA)

The activity of NF-κB in PanCa cells was determined using An EMSA. Binding reaction mixtures (20 μl) containing 3 μl of nuclear extract protein (2-5 μg/μl), 1 μl of poly (dI-dC) in 10 mM Tris and 1 mM EDTA (1 μg/μl), 1 μl of MgCl_2 _(100 mM), 2 μl of 25 mM DTT/2.5% Tween-20, 1 μl of 1% NP-40, 2 μl of 10 × Binding Buffer (100 mM Tris, 500 mM KCl, 10 mM DTT, pH 7.5), 1 μl of IRDye™ end labelled oligo probe (50 nM) (LI-COR Biosciences, Lincoln, NE, USA) and 9 μl of Ultra Pure water were incubated for 30 minutes at room temperature. Proteins were separated by electrophoresis in a native 5% polyacrylamide gel at 4°C in running buffer (12.5 mM Tris borate, 0.25 mM EDTA; pH 8.0), then imaged using the LI-COR Bioscience Odyssey Imaging System according to the manufacturer's instructions. The sequence of the NF-κB-specific probe was 5'-AGT TGA GGG GAC TTT CCC AGG C-3'.

### Western blotting analysis

The MIA PaCa-2 and BxPC-3 cells were treated with metoprolol (100 μM) and ICI118,551 (100 μM) for 24 h. The proteins were then extracted from the treated cells. The supernatant was collected and the total protein concentration was measured using the BCA assay kit (Pierce, Rockford, IL, USA) according to the manufacturer's instructions. The total proteins were used for Western blotting analysis using specific antibodies against caspase-3, caspase-9, Bcl-2, Bax, cyclin D1, cyclin E (Abcam, Cambridge, MA, USA), pERK, ERK, pAkt, and Akt (Cell Signal Technology, Beverly, MA, USA). Thirty μg of total protein each sample was separated by 12% SDS-polyacrylamide gel electrophoresis (SDS-PAGE), and the protein was electrically transferred onto a nitrocellulose membrane (Millipore, Bedford, MA, USA). The membrane was then blocked with TBST (10 mM Tris-HCl, pH 7.4, 150 mM NaCl, 0.1% Tween-20) containing 5% w/v non-fat dry milk and incubated with the primary antibody in TBST at 4°C overnight. The membrane was washed three times and was then incubated with the secondary antibody for 2 h at room temperature. After washing three times for 10 min each with 15 ml of TBST, signallings were visualised by enhanced chemiluminescence (Pierce, Rockford, IL, USA) associated fluorography.

### Statistical analysis

The data were analysed by one-way ANOVA, and by the nonparametric Kruskal-Wallis test. The relationship between β-AR expression and clinical-pathologic character were analysed by Chi-square test. Values were expressed as the mean ± SEM and the differences were considered statistically significant at *P *< 0.05.

## Results

### Expression of β-adrenoceptors in PanCa cell lines and clinical samples

**Figure 1:**
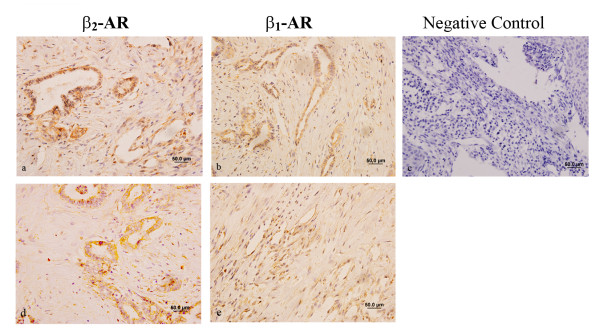
**Expression levels of β_1 _and β_2_-adrenoceptor in the human ductal pancreatic adenocarcinoma cells**. Two representative immunohistochemical staining of PanCa cells presented from 48 pancreatic carcinoma specimen samples (expressions of β_1_-adrenergic receptor [b and e], the expressions of β_2_-adrenergic receptor [a and d] and the negative control [c] in).

Our previous study showed that β_1_- and β_2_-adrenergic receptors were expressed in PanCa cells by Real-time RT-PCR and Western blotting [14]. In the current study, we further determined the expression of β_1_- and β_2_-adrenergic receptors in pancreatic carcinoma specimens using immunohistochemical staining. As shown in Figure 1, PanCa cells in biopsy specimens express bothβ_1_-AR and β_2_-AR on the membrane in a large number of epithelial cells and the β-ARs are localized in the plasma membrane of pancreatic cancer cells, and the representative images are shown in Figure 1. The 48 pancreatic carcinoma specimen samples from 30 males and 18 females include 18 poorly differentiated pancreatic carcinoma samples, 23 moderately differentiated pancreatic carcinoma samples, and 7 well-differentiated pancreatic carcinoma samples. The relationship between β-AR expression and clinical-pathologic character is showed in the Table 1.

**Table 1:** The relationship between β-AR expression and clinical-pathologic character

Clinical and pathologic character	**β**_**1**_**-AR expression score**	**β**_**2**_**-AR expression score**
Pathology		
Well-differentiated (7)	3.2	2.5
Moderately differentiated (23)	4.9	4.3
Poorly differentiated (18)	5.4	5.3

TNM stage		
I phase (7)	1.6	1.5
II phase (9)	3.9	3.4
III phase (19)	5.5*	5.9*
IV phase (13)	7.8*	7.9*

* *P *< 0.05 Analysed by Chi-square test.

### Cell cycle analysis after β-adrenergic antagonist treatment

**Figure 2:**
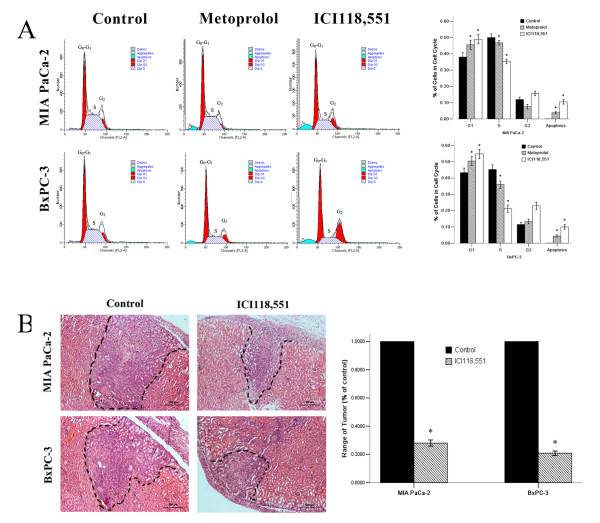
**β-adrenoceptor antagonists regulate cell cycle distribution *in vitro *cultured PanCa cells and limited prevented tumour progression *in vivo***. The cell cycle distribution of PanCa cells in response to β-adrenoceptor antagonists (A), MIA PaCa-2 and BxPC-3 cells were untreated (control) or treated with β-adrenoceptor antagonists: 100 μM metoprolol and 100 μM ICI118,551 for 24 h. β-adrenoceptor antagonists limited tumor development in BALB/c athymic nude mice (B), the black dashed line was used to label the tumor range. H&E staining statistical graph of tumor range showed that the proliferation of PanCa cells was strongly suppressd in renal capsule xenografts after ICI118,551 treatment compared to non-treated cells (*P *< 0.05). *P < 0.05 when compared with controls.

Our early published study showed the optimal concentrations of ICI118,551 and metoprolol for PanCa cell inhibition [14]. In brief, MIA PaCa-2 and BxPC-3 cells were treated with various concentrations from 25 to 200 μM of ICI118,551 and metoprolol for 24 h, the maximal response was obtained with a range of 100 - 200 μM ICI118,551 and metoprolol [14]. In this study, we used 100 μM of β_2_-adrenoceptor antagonist to examine the effect on the cell cycle phase distribution of MIA PaCa-2 and BxPC-3 cells. Based on the data of cell cycle analysis, we found a prolonged and prominent delay in progression from G0-G1 phase after the cells treated with β-adrenoceptor antagonists (Figure 2A) (G0-G1 phase: Control < Metoprolol < ICI118,551, *P *< 0.05), a decrease at the S phase (S phase: Control > Metoprolol > ICI118,551, *P *< 0.05). Moreover, sub-G1 apoptotic compartment was also observed in MIA PaCa-2 and BxPC-3 cells (sub-G1 apoptotic compartment: Control < Metoprolol < ICI118,551, *P *< 0.05). The percentages of G0-G1 phase increased, and the percentages of S phase decreased significantly in the cells exposed to ICI118,551 compared with metoprolol treatment groups (*P *< 0.05). The effect of the ICI118,551 on G_1_/S phase arrest was greater in BxPC-3 cells than in MIA PaCa-2 cells (*P *< 0.05), suggesting a lower sensitivity to β_2_-adrenoceptor antagonist in the cells containing an activating k-ras mutation (Figure 2A).

### Subrenal capsular assay

In the subrenal capsular assay, PanCa cells maintained a solid structure and 3-dimensional growth under the kidney capsule. The implanted tumors could invade the kidney parenchyma, as demonstrated by detection of the tumor cells surrounding the renal glomeruli and tubules. As shown in Figure 2B, the implanted tumors are characterized by malignant growth *in vivo*, including frequent mitosis, neovascularization and invasion into normal tissues. H&E staining results showed that the proliferation of PanCa cells was strongly suppressed in the renal capsule xenografts in mice after ICI118,551 treatment, the range and the depth of invasion was significantly inhibited compared to the non-treated cells (Figure 2B, P < 0.05).

### Cell apoptosis analysis after β-adrenergic antagonist treatment

**Figure 3:**
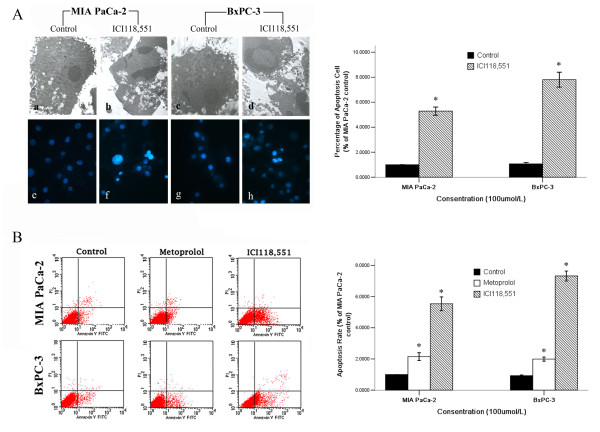
**β-adrenoceptor antagonists induce PanCa cell death**. The morphological features of cell death (necrosis and/or apoptosis) were analyzed by using TEM (A [a-d]×5000). MIA PaCa-2 and BxPC-3 cell induced cell death by ICI118,551 (100 μM) was further confirmed by Hoechst 33342 fluorescent staining (A [e-h]×400), BxPC-3 cells were more sensitive to apoptosis induction than MIA PaCa-2 cells (*P *< 0.05). The apoptotic MIA PaCa-2 and BxPC-3 cells were detected by flow cytometry (B). MIA PaCa-2 and BxPC-3 cells were treated with β-adrenoceptor antagonists (100 μM). Exposure to the β_2_-adrenergic antagonist ICI118,551 significantly increased cell apoptosis compared to the metoprolol group (*P *< 0.05), BxPC-3 cells were more sensitive to apoptosis induction than MIA PaCa-2 cells(*P *< 0.05) (B). *P < 0.05 when compared with controls.

To investigate the morphological features of cellular death induced by β-adrenergic antagonists, MIA PaCa-2 and BxPC-3 cells were analysed by TEM (Figure 3A). Untreated MIA PaCa-2 and BxPC-3 cells exhibited typical ultrastructure that was characterised by a well-preserved plasma membrane, a nucleus with finely granular and uniformly dispersed chromatin, and a cytoplasm containing randomly distributed organelles and electron-dense granules (a and c). When cells were exposed to ICI118,551 for 24 h, unequivocal signs of apoptosis were detected. The morphological changes observed in these cells included a progressive margination and condensation of the chromatin abutting the inner nuclear envelope, nuclear fragmentation and cytoplasm shrinkage (b and d).

In this study, the effect of ICI118,551 was examined in PanCa cells by Hoechst staining (Figure 3A). Figure 3A shows the characteristic appearance of the apoptotic PanCa cells after treatment with ICI118,551 for 24 h. The ICI118,551 treatment resulted in a significant increase in the number of apoptotic cells (f and h in Figure 3A), compared with basal apoptosis levels in untreated controls (e and g in Figure 3A). The percentage of apoptotic cells in the ICI118,551-treated group was statistically different from the untreated control cells (*P *< 0.05).

The percentages of early apoptotic cells increased significantly after treatment with metoprolol (100 μM) and ICI118,551 (100 μM) in MIA PaCa-2 and BxPC-3 cells (Figure 3B). Additionally, exposure to the β_2_-adrenergic antagonist ICI118,551 significantly increased cell apoptosis when compared to the group treated with the β_1_-adrenergic antagonist metoprolol. BxPC-3 cells, which contain the wild-type k-ras gene, were more sensitive to apoptosis induction than MIA PaCa-2 cells, which harbour an activation point mutation in codon 12 of the k-ras gene (*P *< 0.05).

### Detection of ERK1/2 and Akt phosphorylation and NF-κB activation

**Figure 4:**
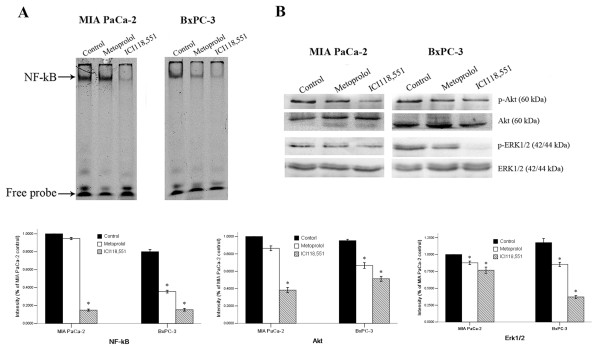
**β-adrenoceptor antagonists limited the activation of NF-κB, ERK, and Akt**. The NF-κB activity in PanCa cells in response to β_2_-adrenoceptor antagonists (**A**) and the phosphorylation state of ERK and Akt in PanCa cells in response to β_2_-adrenoceptor antagonists (**B**) *P < 0.05 when compared with controls.

We next investigated whether the effect of ICI118,551 on MIA PaCa-2 and BxPC-3 cells is associated with the inhibition of NF-κB activation and the induction of ERK, and Akt dephosphorylation. The EMSA and WB results showed that ICI118,551 completely suppressed NF-κB activation and significantly inhibited, ERK1/2 and Akt phosphorylation in MIA PaCa-2 and BxPC-3 cells after 24 h of treatment (Figure 4). We also found that the β_2_-adrenergic antagonists inhibited NF-κB activation, ERK1/2 and Akt phosphorylation more significantly than metoprolol in MIA PaCa-2 and BxPC-3 cells (Figure 4).

### The relationship of β-adrenoceptor antagonist treatment with the expression of caspase-3, caspase-9, Bcl-2, Bax, cyclin D1, and cyclin E

**Figure 5:**
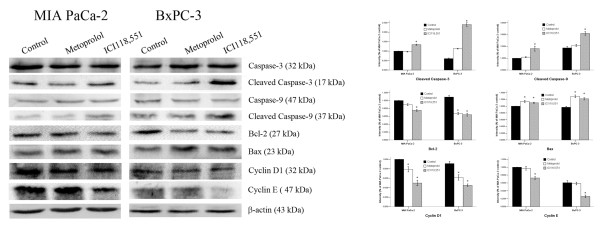
**Expression of caspase-3, caspase-9, Bcl-2, Bax, cyclin D1 and cyclin E in MIA PaCa-2 and BxPC-3 cells in response to β-adrenoceptor antagonists**. A negative control group is also included. The expression of the respective proteins in the whole cell lysates was examined by Western blotting. β-adrenoceptor antagonists increased the level of caspase-3, caspase-9, and Bax, while they negatively affected Bcl-2, cyclin D1, and cyclin E. *P < 0.05 when compared with controls.

The caspase family proteases, caspase-3, caspase-9, Bcl-2, and Bax, have been reported to be involved in apoptosis. In this current study, caspase-3, caspase-9, Bcl-2, and Bax were detected by Western blotting in MIA PaCa-2 and BxPC-3 cells after treatment with metoprolol (100 μM) and ICI118,551 (100 μM) for 24 h. As shown in Figure 5, the cleavage of caspase-3, caspase-9, and Bax increased after treatment with β-adrenergic antagonists compared to the controls; ICI118,551 yielded more prominent bands than metoprolol. However, Bcl-2 decreased after treatment with β-adrenergic antagonists. These results suggested that the β-adrenergic antagonists affect the expression levels of Bax, caspase-3, and caspase-9 in MIA PaCa-2 and BxPC-3 cells.

We also examined the effects of ICI118,551 on the regulatory proteins responsible for cell cycle progression. The protein levels of cyclin D1 and cyclin E were decreased in the two cell lines treated with β-adrenergic antagonists. These results indicate that β_2_-adrenergic antagonists have an effect on some of the cell cycle regulatory proteins responsible for the G_1_/S phase transition and on cell cycle progression (Figure 5).

## Discussion

In this study, we have demonstrated that β-adrenoceptors are expressed in PanCa cells. Our data suggested that the β-adrenergic antagonists were associated with the inhibition of PanCa cell proliferation and the induction of apoptosis and G_1_/S phase arrest. In addition, these effects mediated by the β_2_-adrenergic antagonist were associated with the decreased expression of pERK1/2, pAkt, and the activation of NF-κB. Furthermore, we have found that the β_2_-adrenergic antagonist ICI118,551 significantly increased PanCa cell apoptosis and G_1_/S phase arrest. The effects of ICI118,551 on the rate of apoptosis and on G1/S phase arrest were stronger than the β_1_-adrenoceptor antagonist metoprolol.

The G_1_/S phase transition is a particularly important cell cycle control point. The cyclin D1 is a critical cell cycle regulatory protein and required for the progression through G1 to S phase [17,26]. The growth stimulatory signals in the form of increasing levels of cyclin D1 are received by the cells in the early stage of G_1_, which are then followed by increasing levels of cyclin E at the later stage [26]. ICI118,551 could remarkably down-regulate cyclin D1 and cyclin E expression, indicating that the negatively regulatory effect of the ERK and Akt pathway on the PanCa cell might be associated with the inhibition of NF-κB activation, a decrease in cyclin D1 expression and a delay in the G_1_/S transition, and cell cycle process. Our data has demonstrated that ICI118,551 could disengage the prevention of apoptosis in PanCa cells via suppression of the ERK and Akt pathways and NF-κB activation, and consequently limited the activation of Bax, caspase-9, and caspase-3. The ERK and Akt pathway can phosphorylate Bad, which allows Bcl-2 to form homodimers that result in the generation of an antiapoptotic response. This phosphorylation allows Bcl-2, Bcl-XL, and Mcl-1 to bind Bax and prevent its activation [27-29].

The Ras pathway is commonly altered in PanCa. Activating point mutations in the *K-ras *gene are common in ductal pancreatic carcinomas. NNK induces activating point mutations in the *ras *gene, so it is conceivable that the binding of NNK to β_2_- adrenoceptors may stimulate this pathway via further activation of the Src tyrosine kinase and Ras. The effects of the β_2_-adrenergic antagonist ICI118,551 on the G_1_/S phase arrest and apoptosis are stronger in the BxPC-3 cells (expressing wild-type k-ras) than in the MIA PaCa-2 cells containing a k-ras mutation. Therefore, it suggests that k-ras may be a key molecule involved in the blockage of the β-adrenoceptor resulting in G_1_/S arrest and apoptosis in PanCa cells. Previous study suggested that β_2_-adrenergic receptor antagonist induces cell death by inhibiting the Ras/Raf/Erk1/2 and PI3K/Akt pathways and mitochondrial death pathway [30]. Our results demonstrated that β_2_-adrenergic receptor antagonist induced cell death in MIA PaCa-2 and BxPC-3 cells is independent of the mitochondrial Apaf-1/caspase-9 pathway.

Strategies to block β-adrenoceptors might be used to prevent cancer in smokers and stress patients, and for the prevention of cancer relapse and metastasis after conventional chemotherapy or surgical resection [4,15]. Considering that the β_2_-adrenergic antagonists could be developed as therapeutic and preventive agents, we suggest that understanding the relationship between β_2_-adrenoceptor signalling and G_1_/S phase arrest and apoptosis may provide a new strategic therapy for pancreatic cancer.

## Competing interests

The authors declare that they have no competing interests.

## Authors' contributions

DZ, ZW, MZ, and KG carried out the experimental work, FW provided data analysis, QM provided tumor samples, clinical information, and histopathological analysis, DZ, ZW, QM, FW, and EW designed the study and participated in writing the paper. All authors read and approved the manuscript.

## References

[B1] Jemal A Siegel R Ward E Hao Y Xu J Thun MJ Cancer statistics, 2009 CA Cancer J Clin 2009 59 225 249 10.3322/caac.20006 19474385

[B2] Raimondi S Maisonneuve P Lowenfels AB Epidemiology of pancreatic cancer: an overview Nat Rev Gastroenterol Hepatol 2009 6 699 708 10.1038/nrgastro.2009.177 19806144

[B3] Hidalgo M Pancreatic cancer N Engl J Med 2010 362 1605 1617 10.1056/NEJMra0901557 20427809

[B4] Antoni MH Lutgendorf SK Cole SW Dhabhar FS Sephton SE McDonald PG Stefanek M Sood AK The influence of bio-behavioural factors on tumour biology: pathways and mechanisms Nat Rev Cancer 2006 6 240 248 10.1038/nrc1820 16498446 PMC3146042

[B5] Al-Wadei HA Schuller HM Nicotinic receptor-associated modulation of stimulatory and inhibitory neurotransmitters in NNK-induced adenocarcinoma of the lungs and pancreas J Pathol 2009 218 437 445 10.1002/path.2542 19274673 PMC3372983

[B6] Davis R Rizwani W Banerjee S Kovacs M Haura E Coppola D Chellappan S Nicotine promotes tumor growth and metastasis in mouse models of lung cancer PLoS One 2009 4 e7524 10.1371/journal.pone.0007524 19841737 PMC2759510

[B7] Thaker PH Han LY Kamat AA Arevalo JM Takahashi R Lu C Jennings NB Pena GA Bankson JA Ravoori M Merritt WM Lin YG Mangala LS Kim TJ Coleman RL Landen CN Li Y Felix E Sanguino AM Newman RA Lloyd M Gershenson DM Kundra Vi Lopez-Berestein G Lutgendorf SK Cole SW Sood AK Chronic stress promotes tumor growth and angiogenesis in a mouse model of ovarian carcinoma Nat Med 2006 12 939 944 10.1038/nm1447 16862152

[B8] Sood AK Bhatty R Kamat AA Landen CN Han L Thaker PH Li Y Gershenson DM Lutgendorf S Cole SW Stress hormone-mediated invasion of ovarian cancer cells Clin Cancer Res 2006 2 369 375 10.1158/1078-0432.CCR-05-1698 PMC3141061 16428474

[B9] Wu WK Wong HP Luo SW Chan K Huang FY Hui MK Lam EK Shin VY Ye YN Yang YH Cho CH 4-(Methylnitrosamino)-1-(3-pyridyl)-1-butanone from cigarette smoke stimulates colon cancer growth via beta-adrenoceptors Cancer Res 2005 65 5272 5277 10.1158/0008-5472.CAN-05-0205 15958573

[B10] Schuller HM Neurotransmission and cancer: implications for prevention and therapy Anticancer Drugs 2008 19 655 671 10.1097/CAD.0b013e3283025b58 18594207

[B11] McGraw DW Liggett SB Molecular mechanisms of β_2_-adrenergic receptor function and regulation Proc Am Thorac Soc 2005 2 292 296 10.1513/pats.200504-027SR 16267351 PMC2713324

[B12] Schuller HM Al-Wadei HA Neurotransmitter receptors as central regulators of pancreatic cancer Future Oncol 2010 6 221 228 10.2217/fon.09.171 20146581 PMC2832917

[B13] Weddle DL Tithoff P Williams M Schuller HM Beta-adrenergic growth regulation of human cancer cell lines derived from pancreatic ductal carcinomas Carcinogenesis 2001 22 473 479 10.1093/carcin/22.3.473 11238189

[B14] Zhang D Ma QY Hu HT Zhang M Beta2-adrenergic antagonists suppress pancreatic cancer cell invasion by inhibiting CREB, NF-kappaB and AP-1 Cancer Biol Ther 2010 10 19 29 10.4161/cbt.10.1.11944 20424515

[B15] Schuller HM Mechanisms of smoking-related lung and pancreatic adenocarcinoma development Nat Rev Cancer 2002 2 455 463 10.1038/nrc824 12189387

[B16] Askari MD Tsao MS Schuller HM The tobacco-specific carcinogen, 4-(methylnitrosamino)-1-(3-pyridyl)-1-butanone stimulates proliferation of immortalized human pancreatic duct epithelia through beta-adrenergic transactivation of EGF receptors J Cancer Res Clin Oncol 2005 131 639 648 10.1007/s00432-005-0002-7 16091975 PMC12161179

[B17] McCubrey JA Steelman LS Chappell WH Abrams SL Wong EW Chang F Lehmann B David TM Michele M Agostino T Franca S Massimo L Jorg B Camilla E Alberto MM Richard AF Roles of the Raf/MEK/ERK pathway in cell growth, malignant transformation and drug resistance Biochim Biophys Acta 2007 1773 1263 1284 10.1016/j.bbamcr.2006.10.001 17126425 PMC2696318

[B18] Majidi M Al-Wadei HA Takahashi T Schuller HM Nongenomic beta estrogen receptors enhance beta1 adrenergic signaling induced by the nicotine-derived carcinogen 4-(methylnitrosamino)-1-(3-pyridyl)-1-butanone in human small airway epithelial cells Cancer Res 2007 67 6863 6871 10.1158/0008-5472.CAN-07-0483 17638897

[B19] Vargiu P De Abajo R Garcia-Ranea JA Valencia A Santisteban P Crespo P Bernal J The small GTP-binding protein, Rhes, regulates signal transduction from G protein-coupled receptors Oncogene 2004 23 559 568 14724584 10.1038/sj.onc.1207161

[B20] Luttrell LM Ferguson SS Daaka Y Miller WE Maudsley S Della Rocca GJ Lin FT Kawakatsu H Owada K Luttrell DK Caron MG Lefkowitz RJ β-Arrestin-dependent formation of β_2 _adrenergic receptor-src protein kinase complexes Science 1999 283 655 661 10.1126/science.283.5402.655 9924018

[B21] Hirsch E Ciraolo E Ghigo A Costa C Taming the PI_3_K team to hold inflammation and cancer at bay Pharmacol Therapeut 2008 118 192 205 10.1016/j.pharmthera.2008.02.004 18420279

[B22] Zhang D Ma Q Shen S Hu H Inhibition of pancreatic cancer cell proliferation by propranolol occurs through apoptosis induction: the study of beta-adrenoceptor antagonist's anticancer effect in pancreatic cancer cell Pancreas 2009 38 94 100 10.1097/MPA.0b013e318184f50c 19106745

[B23] Hu HT Ma QY Zhang D Shen SG Han L Ma YD Li RF Xie KP HIF-1alpha links beta-adrenoceptor agonists and pancreatic cancer cells under normoxic condition Acta Pharmacol Sin 2010 31 102 110 10.1038/aps.2009.181 20037603 PMC4002695

[B24] Gardner-Thorpe J Ito H Ashley SW Whang EE Differential display of expressed genes in pancreatic cancer cells Biochem Biophys Res Commun 2002 293 391 395 10.1016/S0006-291X(02)00237-1 12054612

[B25] Chen ZB Liu C Chen FQ Li SY Liang Q Liu LY Effects of tobacco-specific carcinogen 4-(methylnitrosamino)-1-(3-pyridyl)-1-butanone (NNK) on the activation of ERK1/2 MAP kinases and the proliferation of human mammary epithelial cells Environ Toxicol Pharmacol 2006 22 283 291 10.1016/j.etap.2006.04.001 21783722

[B26] Foster I Cancer: A cell cycle defect Radiography 2008 14 144 149 10.1016/j.radi.2006.12.001

[B27] Leicht DT Balan V Kaplun A Singh-Gupta V Kaplun L Dobson M Guri T Raf kinases: Function, regulation and role in human cancer Biochim Biophys Acta 2007 1773 1196 1212 10.1016/j.bbamcr.2007.05.001 17555829 PMC1986673

[B28] Weyden L Adams DJ The Ras-association domain family (RASSF) members and their role in human tumourigenesis Biochim Biophys Acta 2007 1776 58 85 17692468 10.1016/j.bbcan.2007.06.003 PMC2586335

[B29] Chambard JC Lefloch R Pouysségur J Lenormand P ERK implication in cell cycle regulation Biochim Biophys Acta 2007 1773 1299 1310 10.1016/j.bbamcr.2006.11.010 17188374

[B30] Herr I Debatin KM Cellular stress response and apoptosis in cancer therapy Blood 2001 98 2603 2614 10.1182/blood.V98.9.2603 11675328

